# Resilience at the Transition to Agriculture: The Long-Term Landscape and Resource Development at the Aceramic Neolithic Tell Site of Chogha Golan (Iran)

**DOI:** 10.1155/2015/532481

**Published:** 2015-08-06

**Authors:** S. Riehl, E. Asouti, D. Karakaya, B. M. Starkovich, M. Zeidi, N. J. Conard

**Affiliations:** ^1^Institute for Archaeological Sciences, University of Tübingen, Rümelinstraße 23, 72070 Tübingen, Germany; ^2^Tübingen Senckenberg Center for Human Evolution and Palaeoenvironment, Rümelinstraße 23, 72070 Tübingen, Germany; ^3^Department of Archaeology, Classics and Egyptology, University of Liverpool, 12-14 Abercromby Square, Liverpool L69 7WZ, UK; ^4^Abteilung für Ältere Urgeschichte und Quartärökologie, Institut für Ur- und Frühgeschichte und Archäologie des Mittelalters, Universität Tübingen, Schloss Hohentübingen, 72070 Tübingen, Germany

## Abstract

The evidence for the slow development from gathering and cultivation of wild species to the use of domesticates in the Near East, deriving from a number of Epipalaeolithic and aceramic Neolithic sites with short occupational stratigraphies, cannot explain the reasons for the protracted development of agriculture in the Fertile Crescent. The botanical and faunal remains from the long stratigraphic sequence of Chogha Golan, indicate local changes in environmental conditions and subsistence practices that characterize a site-specific pathway into emerging agriculture. Our multidisciplinary approach demonstrates a long-term subsistence strategy of several hundred years on wild cereals and pulses as well as on hunting a variety of faunal species that were based on relatively favorable and stable environmental conditions. Fluctuations in the availability of resources after around 10.200 cal BP may have been caused by small-scale climatic fluctuations. The temporary depletion of resources was managed through a shift to other species which required minor technological changes to make these resources accessible and by intensification of barley cultivation which approached its domestication. After roughly 200 years, emmer domestication is apparent, accompanied by higher contribution of cattle in the diet, suggesting long-term intensification of resource management.

## 1. Introduction

*Explaining the Beginnings of Agriculture: An Accumulated Record*. Since the first half of the 20th century the importance of agriculture in the history of humankind has been continuously emphasized along with a steady development of models on the natural conditions and cognitive circumstances under which the neolithization process took place.

From a bioarchaeological perspective many questions on the associated changes seem to be well understood. During the last decades identification criteria for distinguishing wild and domesticated plant species have improved (e.g. [1]). In some cases it is even possible to distinguish between gathered and cultivated wild assemblages through consideration of grain size and the presence of weed species [2–4]. Similar methodological developments in the study of faunal remains enabled novel insights into the emergence of animal domestication (e.g. [5, 6]). Radiocarbon dating of morphologically domesticated species and DNA analyses of modern species allowed the localization and timing of the first domesticated plant and animal species [7–10]. Archaeobotanical research resulted in the abandonment of the hypothesis of a single core area for the origins of agriculture within the Fertile Crescent [11, 12]. The accumulated record of archaeobotanical assemblages with different proportions of wild and domesticated species supported the model of protracted domestication lasting up to 2000 years ([2, 11, 13, 14], but see also [15] for a different opinion). The term “protracted domestication” is often used with absolute chronological designations, for example, marking the time range between large-scale systematic gathering of wild cereals at Ohalo II around 23.000 BP and the appearance of the first domesticated species during the PPNB [13], contrasted with possible “rapid domestication” resulting from the intentional selection of domesticated phenotypes which may take place within a few cereal generations [16]. We, however, consider the qualitative connotation of the term to be less arbitrary and use “protracted” as a relative term, indicating an evolutionary process with phases of stasis or even reversal. With such an understanding “protracted domestication” can occur equally within a process of 1.000 or 15.000 years.

Climatic fluctuations following the end of the last glacial maximum have been comprehensively investigated and favored by some to represent a key catalyst for the development of agriculture under a combined contribution of changes in resource availability and demographic pressures [17–22]. Others use global palaeoclimate records to argue for the mandatory beginnings of agriculture during the Holocene, by linking them to the rate of innovation of subsistence technology or subsistence-related social organization [23–25]. With some exceptions [26], such generalizations rarely incorporate the regional climatic diversity that is fundamental in considerations of agricultural development during later periods (e.g. [27]), and that is evidenced in the palaeoclimatic record (e.g. [28–30]). One obstacle for integrating local palaeoclimate proxies and the archaeological record is the considerable distance of some archaeological sites from the locations of major palaeoclimate archives. In the archaeological record climatic fluctuations are often difficult to pinpoint, as the nature of the palaeoenvironmental evidence is superimposed by human activity. In addition, climate-relevant parameters in palaeoclimatic archives often show the character of creeping normalcy, thus would not necessarily have been recognized by ancient people, or may have allowed for adaptive measures provoking further environmental change through anthropogenic impacts [31].

Alongside the unknown mode of ancient people's perceptions that shaped their individual interactions with the environment goes the socio-natural development of the human species itself that has been less frequently included into neolithization models. What has earlier been considered as the coevolution of domesticates and human subsistence strategies [32], has been further developed by integrating insights from cognitive science [33]. In focusing on the biological aspects of* Homo sapiens* evolution, the form and functionality of the human brain have often been considered to have changed little, although the cognitive and cultural faculties as tangible through the archaeological record have changed considerably over time (see also [34]).

This paper focuses specifically on two aspects of the archaeological record that are particularly relevant to the investigation of agricultural origins: archaeobotanical and zooarchaeological assemblages. Our goal is to address the major epistemological issue of building broad-scale, cross-regional generalizations on the basis of a fragmentary record.

An impediment to the investigation of the local, long-term transitional developments from cultivating wild and later domesticated species, is the generally short-term occupation at aceramic Neolithic sites, in most cases spanning less than 2.000 years. The nature of the evidence on the beginnings of agriculture is therefore accumulated from disparate locations across the Fertile Crescent. Emerging problems from adding up snapshots from sites at different geographic locations with the goal of reconstructing a developmental sequence relate to the above described differences in local climatic and environmental conditions, as well as in socio-cultural identities, landscape perceptions and associated strategies of the prehistoric populations (see discussion in [35, 36]). The shorter the archaeological sequence at a particular site, the greater likelihood that our generalizations on evolutionary and developmental processes capture only a limited picture.

The long-term archaeobiological record from the aceramic Neolithic site of Chogha Golan, representing more than 2.000 years, allows the investigation of a multifactorial interplay of humans with their environment, including adaptive cycles at the transition to agriculture.

**Figure 1:**
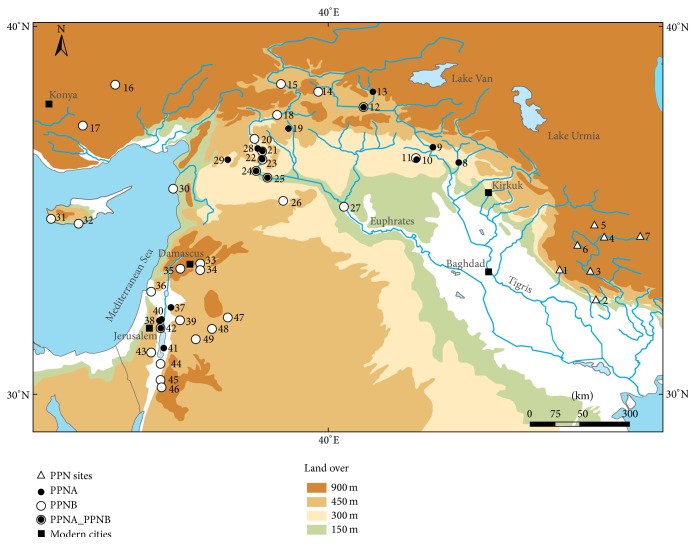
Chogha Golan (1) and its geographic position within the Fertile Crescent; (2) Ali Kosh, (3) Chia Sabz, (4) Ganj Dareh Tepe, (5) Sheikh-e Abad, (6) Jani, (7) Tepe Abdul Hosein, (8) M'lefaat, (9) Nemrik, (10) Qermez Dere, (11) Magzalia, (12) Körtik Tepe, (13) Hallan Cemi, (14) Cayonu, (15) Cafer Hoyuk, (16) Asikli Hoyuk, (17) Can Hasan III, (18) Nevali Cori, (19) Göbekli Tepe, (20) Akarcay Tepe, (21) Djade, (22) Halula, (23) Jerf al Ahmar, (24) Mureybet, (25) Abu Hureyra, (26) El Kowm I & II, (27) Bouqras, (28) Abr, (29) Qaramel, (30) Tell Ras Shamra, (31) Kissonerga, (32) Parekklisha-Shillourokambos, (33) Tell Ghoraifé, (34) Tell Aswad, (35) Tell Ramad, (36) Yiftah'el, (37) Iraq ed Dubb, (38) Gilgal, (39) ‘Ain Ghazal, (40) Netiv Hagdud, (41) Dhra, (42) Jericho, (43) Nahal Hemar, (44) Wadi Fidan, (45) Beidha, (46) Basta, (47) Dhuweila, (48) Azraq 31, (49) Wadi Jilat 7; PPN is applied to Iranian sites, because PPNA and PPNB have additional cultural connotations that do only apply to sites in the western and northern part of the FC.

*Chogha Golan and Its Significance for Understanding the Neolithization Process*. The aceramic tell site of Chogha Golan is situated at the lower ranges of the Central Zagros Mountains, about 30 km north of Mehran (Amirabad plain/Ilam Province/Iran) (Figure 1). Starting in 2009 the site was excavated by the Tübingen-Iranian Stone Age Research Project (TISARP) and the Iranian Center for Archaeological Research [37, 38].

Aceramic sites in western Iran have been poorly documented so far, and our knowledge of prehistoric populations in this area derives mainly from surveys and excavations undertaken in the 1950s and 1960s [39–41]. Therefore little is known about the archaeobotany and early human subsistence strategies in the region, including the emergence of plant cultivation and agriculture [4, 42–44]. Animal domestication is more well-studied in the region (e.g., [5, 6, 45–53]), though more work remains to be done.

**Figure 2:**
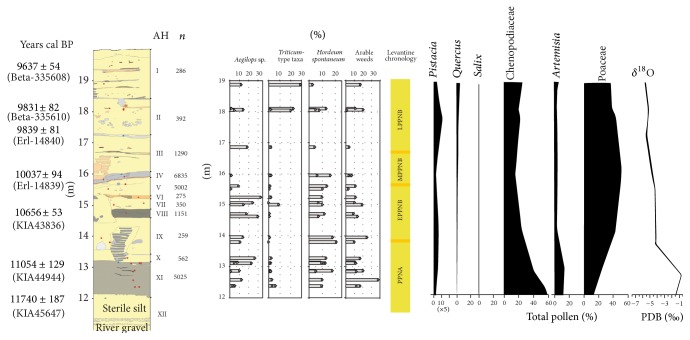
Stratigraphic profile from Chogha Golan with AMS dates in years cal BP (locations of dated samples indicated with blue dots in the profile) and archaeological horizons (AH) in Roman numbers. Percentages of taxa and groups of taxa relevant for the development of cultivation and domestication in each sample based on the total of all identifications from each AH (location of samples are indicated with red dots in the profile):* Aegilops* sp. (goat-grass),* Triticum*-type taxa: agglomeration of different* Triticum* taxa (including* Triticum boeoticum/dicoccoides* grains and glume bases, free-threshing wheat type rachis internodes and unidentified Triticum taxa),* Hordeum spontaneum* (wild barley), probable arable weeds acc. to Willcox [3] including* Trigonella* sp.,* Silene* sp.,* Reseda luteola*,* Ornithogalum/Muscari*,* Medicago radiata*,* Malva *sp.,* Lithospermum *sp.,* Heliotropium *sp.,* Gypsophila *sp.,* Galium *sp.,* Fumaria *sp.,* Erodium *sp.,* Coronilla *sp.,* Centaurea *sp. and* Adonis *sp.; (*n*) number of seed and chaff records identified in each horizon. The yellow bar indicates the equivalent Levantine cultural chronology. The palynological and stable oxygen isotope data (right) represent the relevant sequences of Lake Zeribar record extracted from [55].

Excavations at Chogha Golan revealed 8 m of deposits consisting of 11 archaeological layers (AH XI–I) and one geological horizon at the lowest level of the stratigraphic sequence spanning in all more than 2.000 years. The profile of the deep sounding has been consistently dated, with 17 AMS dates ranging from roughly 11.800 BP (geological horizon) to ca. 9.600 BP (AH I) (Table 1, Figure 2). With the earliest date at the end of the Younger Dryas, Chogha Golan represents one of the oldest aceramic Neolithic sites in Iran, together with Sheikh-e Abad [54]. Most of the archaeological sediments derive from a deep sounding measuring between five and one square meter into a depth of 8 meters. The different archaeological horizons contain ochre-painted plaster floors and mud brick walls interspersed with midden deposits that have been interpreted as the contemporary architecture and associated occupation debris of the site's inhabitants.

The extraordinary richness of the archaeological sediments in artefacts and bioarchaeological remains at Chogha Golan permits a high-resolution investigation of the development of the cultural sequence, subsistence behaviors and the local environment. Large quantities of lithic debitage demonstrate the intensive nature of the activities of the prehistoric community.

The development of the lithic industry over the sequence of 11 archaeological layers is relatively indistinct and may indicate relative stability in tool usage. All layers can be characterized by the systematic production of large numbers of bladelets and tools made on bladelets [38, 56]. The assemblage contains a large component of non-retouched bladelets. The tool diversity from Chogha Golan is relatively low, and most tools appear to have been used for cutting or perforating, with limited indications of scraping and harvesting. The amount of sickle blades with evidence of use wear is also consistently low throughout the archaeological horizons [56]. The comparison of the lithic assemblages at Chogha Golan with other early Neolithic sites in the Zagros highlands and lowlands reveals similarities which place these sites within a cultural and behavioral group associated with the Mlefaatian industry which persisted for almost 2000 years (cf. [57, 58]).

The site's inhabitants routinely exploited locally available raw materials as well as a very low percentage of imported obsidian. In archaeological horizon II and subsequently AH I obsidian occurs for the first time and comprises approximately 0.5% of the lithic material [56]. The presence of obsidian in the upper layers of Chogha Golan fits the diachronic trend of the region of increased obsidian finds around 9600–9500 cal BP [58]. At other sites in the region obsidian has been found to originate from eastern Anatolia [59, 60] suggesting prehistoric mobility patterns dating from as early as the Palaeolithic (cf. [61]).

The first appearance of mudbrick walls, plaster floors and associated ground stone implements such as mortars, pestles and grinding slabs is recorded for AH X. Mortars and grinding slabs were at times found embedded in structure floors showing that they were permanent fixtures in the architecture of the site, while other mortars and grinding slabs were mobile [38, 62]. Although the first appearance of inhabited mudbrick structures with* in situ *preserved ground stone implements are from AH X, most of the mortars, grinding slabs and pestles were recovered from AH III onwards, and were likely used for processing foods. The available data, however, show an emphasis on pounding and little emphasis on grinding. This tendency could be related to the low volume of sediments excavated thus far, rather than an emphasis on pounding activities during the upper occupation. The presence of asphalt and pigments on ground stone tools also demonstrate that they were not exclusively used for food-processing activities. In fact, the available records fit the observation that ground stone tools were often multifunctional devises during the aceramic Neolithic.

A total of 62 clay small objects from stratified deposits were recovered during the course of the 2009 and 2010 field seasons at Chogha Golan. Most clay objects are from ash and midden deposits. They are found with debris from daily activities and are not limited to obvious ritual contexts. Chogha Golan has provided evidence for the use of clay from the 10th millennium cal. BC onward. The clay usage in Chogha Golan is first evidenced by small and simply formed objects. The usage of clay increases through the sequence, probably reflecting social, economic and cultural development. Excavators recovered 10 clay animal figurines. While an unknown animal head and a goat horn were found from AH VI and III respectively, most of the animal figurines representing sheep/goat, cattle and pig were recovered from AH II and I.

Preliminary results of the ongoing archaeobotanical analyses suggest that cultivation of wild plants in the Zagros started equally early as at other places in the Fertile Crescent [4, 63], but here we provide a more detailed picture of the changing economic and landscape practices of the Neolithic inhabitants of Chogha Golan, and their interplay with environmental and cultural shifts. Our aim is to contribute to the ongoing scientific debate concerning the socio-cultural, economic and environmental conditions that enabled the slow pace of the development of cultivation and herding practices and the transition to agriculture.

*The Modern and Early Holocene Environment of the Region*. The present-day climate of Iran is marked by extreme continental conditions (i.e., cold winters and hot, dry summers); the observed mesoclimatic heterogeneity is attributed to the region's complex topography. The strong north-south contrast in atmospheric pressure is due to Iran's position between summer dominating north-east trade winds and the winter dominating westerlies [65]. Chogha Golan is located in a region receiving today between 100–200 mm of mean annual precipitation, while mean annual temperatures range between 20–25°C. More important for considerations of past environmental conditions, however, is that calculated surplus and deficits of precipitation vary considerably over relatively short distances, particularly near the Elburz and Zagros ranges. According to the mapping of surplus and deficits of precipitation, Chogha Golan is situated in an area with more than 800 mm annual deficit in rainfall, but in less than 25 km distance from this region surpluses of 100–400 mm annual precipitation have been calculated (see [65, p. 76]). Considering that isohyets may have shifted with past climatic fluctuations, environmental conditions at Chogha Golan may equally have been very variable throughout time.

**Figure 3:**
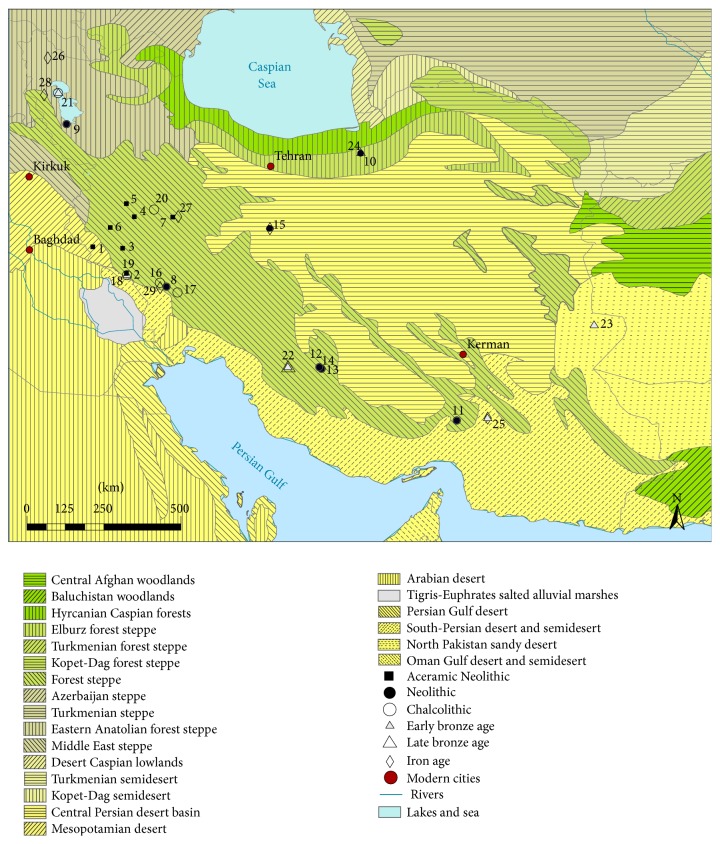
Ecotopes in Iran with archaeobotanically investigated sites: (1) Chogha Golan, (2) Ali Kosh, (3) Chia Sabz, (4) Ganj Dareh Tepe, (5) Sheikh-e Abad, (6) Jani, (7) Tepe Abdul Hosein, (8) Jaffarabad, (9) Tepe Hasanlu, (10) Tepe Musiyan, (11) Tepe Yahya, (12) Tall-e Mushki, (13) Tall-e Jari, (14) Tall-e Bakun, (15) Tepe Sialk, (16) Bendebal, (17) Sharafabad, (18) Tepe Farukhabad, (19) Tepe Sabz, (20) Godin Tepe, (21) Tappeh Gijlar, (22) Malyan, (23) Shahr-i Sokhta, (24) Tepe Hissar, (25) Konar Sandal, (26) Bastam, (27) Nush-i Jan, (28) Qal'eh Ismail Aqa, (29) Susa-Ville Royale.

The modern ecotope around Chogha Golan can be characterized as a desert type vegetation zone or Mesopotamian steppe bordering the* Acacietea* classes of the Sudanian flora as defined by Zohary (Figure 3; [66]). The region is, however, highly diversified hosting geobotanical units ranging from steppe-forests to desert vegetation, comprising elements of the Sudanian and sub-Sudanian flora as well as of the Saharo-Arabian desert vegetation, Irano-Turanian steppe, and Kurdo-Zagrosian steppe-forest sensu Zohary [66]. Characteristic plant associations include the* Acacietea flavae iranica*,* Anabasetea articulatae*,* Artemisietea herbae-albae mesopotamica*,* Pistacia-Amygdalus* steppe forest and* Quercetea brantii* xerophilous deciduous steppe-forest.

Another important landscape unit that is closely associated with Chogha Golan is the Konjan Cham River which is located 200 m from the site, and which would have provided suitable conditions for the growth of vegetation requiring alluvial wetland habitats.

Considering pollen diagrams in more detail to inform about late Quaternary vegetation, extant Lake Mirabad (800 m asl, [67]) is, the closest palaeovegetation proxy archive to Chogha Golan, at only 140 km away. Unfortunately the only palynological analysis available from this location was conducted several decades ago, and is thus lacking a highly resolved chronology [68]. There is however one radiocarbon date (10.370 ± 120 BP) from the lowest core level placing the sequence into the Holocene. According to the authors, the vegetation development reflected in the diagram is very similar to that of the better investigated and dated core from Lake Zeribar (1.285 m asl, 240 km north of Chogha Golan, [55, 69]; see also Figure 2). Palaeoclimate proxy archives are also available from Lake Urmia (1.270 m asl, [70, 71]) 500 km to the north of Chogha Golan (490 m asl).

For the Late Glacial/Early Holocene transition most researchers propose that temperatures were high, therefore so were evaporation rates. These factors, combined with low precipitation led to low lake levels, water salinization and scattered occurrences of a very thin arboreal cover [55, 72]. El-Moslimany has suggested that the high percentages of Chenopodiaceae and* Artemisia* pollen, generally interpreted as indicators of dry steppe habitats and a cold-arid climate, do not necessarily indicate low annual precipitation, but instead a highly seasonal climate with cold winters and hot, dry summers [69].

At the onset of the early Holocene a rise in temperatures and a change to freshwater lake conditions as recorded in the shift in* Najas* species is indicated at Lake Urmia. Unstable and variable water levels are superimposed on generally low lake levels as indicated by the botanical macrofossils [72]. Some researchers have deduced changes in patterns of seasonality. According to Stevens et al. [55], summer rainfall or at least reduced summer drought occurred, while El-Moslimany [69] suggests that changes in seasonality resulted in the dominance of Poaceae pollen and the initial increase in arboreal pollen. Most grasses thrive in environments characterized by summer rainfall, while low pollen production is apparently due to reproductive strategies related to the summer-dry climate [69]. Upland vegetation shifted to a* Pistacia*-deciduous oak semi-arid grassland as low temperatures and aridity ameliorated [55], while winter-dominated precipitation after 10.000 BP is also suggested by low *δ* ^18^O values observed at Lake Mirabad [73].

Differences in the pace of Holocene expansion of oak within the Fertile Crescent from its refugia during the Late Glacial Maximum have been already outlined by Bottema and van Zeist [68, 74–77]. While oak in the Levant is represented with more than 20% in the pollen diagrams during the Younger Dryas and immediately increases with the beginning of the Holocene, it increases only some thousand years later at Lake Van and Lake Zeribar [78]. There is, however, a strong increase in Poaceae, concurrently with the decrease of Artemisia and Chenopodiaceae values at the end of the Younger Dryas event, supported by the *δ* ^18^O record of Lake Zeribar. However, precipitation levels are interpreted to have been low by comparison to the Mediterranean Levant.

Roberts has suggested that human activities such as vegetation burning and woodcutting might have played a role in the early Holocene delay of woodland expansion in the Zagros region [79]. The discovery of charred plant remains in Lake Zeribar sediments brings a new argument to this discussion. Charred particles of herbaceous plants in all sediment cores dating from the Pleniglacial to the present indicate that occasional fires occurred in the wider region, whether these were natural and/or anthropogenic is a matter of debate. Wasylikowa has interpreted the increased frequency of* Plantago lanceolata*-type pollen from about 10.000 cal yr BP as an indicator of the spread of this species in steppe plant communities due to vegetation disturbance by hunters and/or herders [30]. Asouti and Kabukcu [80] have proposed that early Holocene vegetation across the hilly flanks of the Irano-Anatolian region of Southwest Asia was dominated by semi-arid grasslands. The sparse arboreal vegetation was dominated by insect-pollinated Rosaceae associated with erratic pollen producers, such as* Pistacia* and deciduous oak shrubs. The delayed establishment of deciduous woodlands was due to the very rapid response of grasses to the abrupt increase in precipitation values, which provided them with the competitive advantage over trees in seedling establishment. Contrasting with the Levantine littoral, the establishment of woodland vegetation on the mid to low elevation slopes and the plateaus of the Irano-Anatolian region became possible only with the widespread adoption of woodland management strategies and caprine herding. Such practices controlled the growth of grasslands thus enabling intensively managed woodland species such as* Pistacia* and deciduous* Quercus* to form widespread mature woodland pastures [80].

## 2. Materials and Methods

### 2.1. Seed and Chaff Remains

During the excavations in 2009 and 2010 more than 700 sediment samples were recovered and manually floated using 200 *μ*m sieves, of which 45 archaeobotanical samples containing almost 32.000 carbonized seed and chaff remains have been analyzed to date. The mean volume of the sediment samples was 10 liters and find densities of seed and chaff remains in the different archaeological horizons were highly variable, ranging from very low find densities of 10–20 items per liter sediment in the two uppermost layers AH I and AH II to extremely high find densities of 296 items per liter sediment in AH IV (see also Figure 11). The mean find density of all 11 archaeological horizons was 65 seed and chaff remains per liter sediment which is high compared to contemporaneous sites in the Fertile Crescent, such as Tell ‘Abr or Jerf el Ahmar (3 items/liter), Dja'de (5 items/liter) and Tell Qaramel (7 items/liter) [81, 82]. Even in comparison with later periods when settlements are based on fully established agriculture, such as the Bronze Age settlements in Syria [83] the find densities for seeds and chaff remains at Chogha Golan appear to be extremely high. This can be explained by several factors, including a general good preservation for this type of plant remains and the specific contextual situation, that is, their preservation mostly in midden (refuse) contexts, but is also related to the fine stratigraphic excavation of the layers and subsequent careful hand flotation.

All the samples derive from the deep sounding and do not allow a detailed consideration of context. However, an advantage is the long-term sequence of well-dated deposits and the direct comparability of the bioarchaeological remains obtained from the same samples.

Analysis of the seeds was conducted using a Leica GZ6 binocular microscope at the archaeobotanical laboratory of the Institute of Archaeological Science, Tübingen University, based on morphological criteria and the use of the botanical reference collection. In total, 110 botanical taxa were identified (Table 2). Basic numeric methods, such as percentage proportions of taxa and taxa groups and the ubiquity analysis were applied to compare the assemblages of the different archaeological horizons.

### 2.2. Wood Charcoal

Anthracological analyses are ongoing at the Archaeobotany Laboratory of the Department of Archaeology, Classics and Egyptology, University of Liverpool. To date 45 anthracological samples have been analyzed from horizons AH XI and AH IX-I amounting to 1209 wood charcoal particles >2 mm. Judging from the small size of the retrieved charcoal particles (fragments >4 mm were only very occasionally present in the samples) the absence of fresh edges and the relatively low charcoal densities recovered from each archaeological horizon (Figure 11), it can be assumed that, on the whole, the anthracological assemblage had been subjected to a relatively high degree of post-depositional fragmentation prior to field sampling. This appears to be the case with the charcoal macro-remains retrieved from horizon AH VII, and especially from the latest sampled horizon AH I, both of which contained floors and other intensively maintained architectural features. It should also be noted that overall charcoal particles are very well preserved as indicated by the low number of unidentifiable fragments (ranging between 0–7 fragments per sample).

Depending on their size and shape individual charcoal fragments were fractured by hand or with a single edge razor blade across the transverse plane. Where applicable and necessary (also considering the small size of the examined fragments), the tangential and radial longitudinal planes were also obtained. Fresh charcoal surfaces were suspended in a sand bath and examined using high-power, epi-illuminating, dark-field microscopy (magnifications ×40, ×50, ×100, ×200, ×500). Comparative reference materials consulted for wood charcoal identification include a modern charcoal reference collection from Southwest Asia, alongside published wood anatomical descriptions [84–87]. In total, 14 charcoal taxa have been identified. Preliminary results (raw/percentage fragment counts and sample presence) from the samples and size fractions already analyzed are presented in Table 3, tabulated by archaeological horizon.

### 2.3. Faunal Remains

While the archaeobotanical remains studied so far comprise only approximately 6% of the entire assemblage, about one fourth of the faunal remains from the deep sounding sequence have been analyzed, excluding microfaunal remains. We recorded over 5.800 specimens, 1.620 of which were identifiable to species or body class and element. Unidentifiable fragments are recorded in order to document taphonomic processes on the materials. In relation to the archaeobotanical remains, the faunal remains are under-represented. This might be due to bone loss from taphonomic factors, or site use activities that concentrated faunal remains in parts of the tell that are unexcavated.

Faunal specimens were identified using the comparative collection in the Institute for Archaeological Sciences at the University of Tübingen. Documentation of the faunas follows standard zooarchaeological methods and counting units (e.g., [88–92]). All specimens were counted, weighed, and identified to species or body class, element, and portion of element. We recorded human and non-human taphonomic damage when available, as well as information to determine age. Typically, number of identified specimens (NISP) is the preferred counting unit for faunal assemblages [88, 93, 94]. However, during excavation, some horizons were fully (IV, V, VIII, X, XI) or partially (II, III, VI, VII, IX) wet screened, while others were not (I). Because of this, and the potential for NISP to underestimate small taxa in layers that were not wet screened, an alternative method based on weight is used to quantify the remains. Many authors have argued that bone weight represents a more accurate measure of meat mass because it corrects for differences in animal body size, and is less affected by fragmentation [88, 93, 94]. This is particularly true if bones are not mineralized and do not have surface concretions, and if the taphonomic conditions are similar throughout the sequence, which is the case at Chogha Golan.

For the data analysis we categorize individual taxa into groups based on body size and predator evasion tactics (following [95]), because the assemblage considered here is comparatively small. The groups include four sizes of ungulates, small carnivores, small fast, and small-slow moving animals (Table 4). This allows for less diagnostic remains (e.g., medium ungulates) to be included in the analysis.

### 2.4. Stable Carbon Isotope Data

Stable carbon isotope ratios were measured on 159 wild-type barley grains from the site. Measuring stable carbon isotopes in archaeobotanical remains is an established method for identifying past environmental conditions for plant growth in arid and semi-arid environments, as *δ* ^13^C values in cereals provide a drought stress signal when the amount of water received during the grain-filling period is low [98–100]. Taking knowledge of intra-sample variability of *δ* ^13^C into account, a target of minimum measurements of six individual grains per archaeobotanical sample was taken. Well-developed grains were chosen to guarantee the exclusion of *δ* ^13^C values that would not reflect the average growing conditions, such as values obtained from immature grains. To eliminate inputs from sedimentary carbonate, the barley grains were reacted with 0.5 M HCl before measurement. The measurements were carried out at the Institute of Geosciences of the University of Tubingen, Germany on a FinniganMAT252 gas source mass spectrometer with a ThermoFinnigan GasBench II/CTC Combi-Pal autosampler.

The common standard of *δ* ^13^C VPDB (Vienna Peedee belemnite ‰) was applied to the measurements of ^13^C/^12^C ratios to calculate *δ* ^13^C in the barley grains. Changes in atmospheric CO_2_ concentration (*δ* ^13^C air) over time were considered by calibrating the *δ* ^13^C from ancient barley into Δ^13^C values using the approximation AIRCO2_LOESS [101].

## 3. Results

### 3.1. Long-Term Subsistence Development

#### 3.1.1. The Carpological Remains

While 110 seed taxa are recorded for the entire assemblage, only up to 38% of these occur simultaneously in one of the archaeological horizons. Some of the taxa are represented in high numbers, such as the small-seeded Poaceae and Fabaceae, goatgrass and barley and thus characterize the different archaeological layers. Only 16 taxa occur throughout all the 11 archaeological layers, almost exclusively consisting of grasses and pulses (compare frequencies in Table 2).

**Figure 4:**
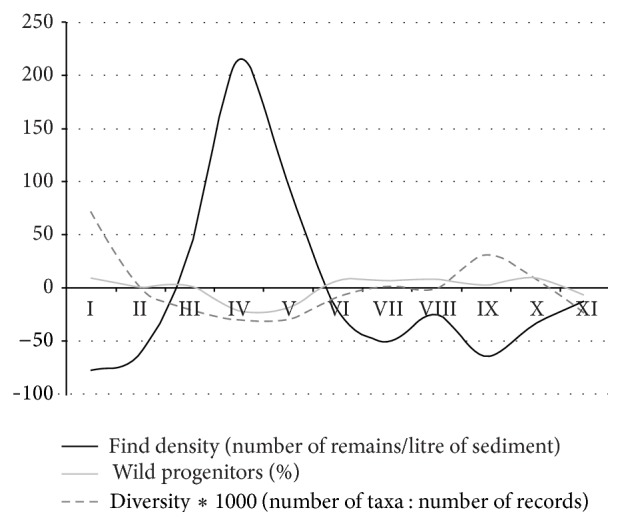
Find density, diversity and percentage of wild progenitor species of crops (all large-seeded grasses and pulses that were domesticated in the Neolithic period, i.e., mainly taxa of* Triticum*,* Hordeum*, and* Lens*) shown in their deviation from the mean represented by the *x*-axis.

The overall diversity, calculated as a ratio of the number of taxa and the number of records, was highest in layer I and IX which may indicate a bias, because these two layers were the poorest as concerns record numbers. The find density, that is, the number of records per liter of sediment, was highest in AH IV and V which is mostly due to a large number of small-seeded grasses in these two layers and correlates with low percentages of wild progenitor species of modern crops (Figure 4).

**Figure 5:**
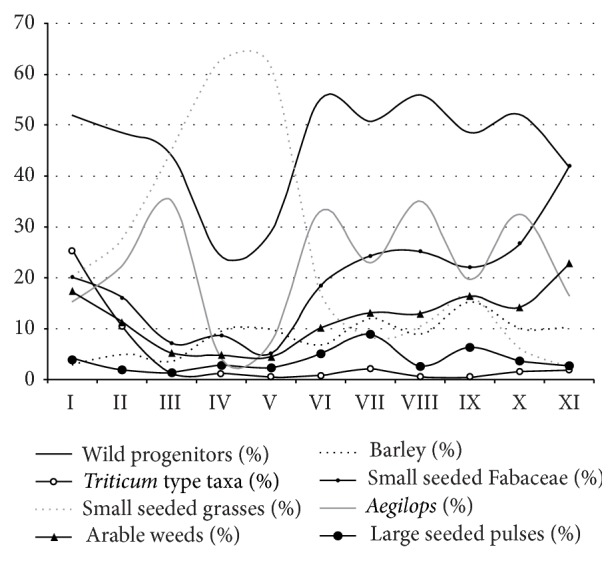
Proportions of the main taxa groups throughout the stratigraphic sequence; Triticum type taxa and barley include wild and domesticated taxa; total number of records on which percentage proportions are based for each archaeological horizon: I: 327, II: 1022, III: 2309, IV: 8875, V: 6584, VI: 1628, VII: 1298, VIII: 1550, IX: 614, X: 942, XI: 6621.

The main taxa groups show distinct developments over the stratigraphic sequence (Figure 5). Large-seeded grasses such as goatgrass (*Aegilops* sp.), the genome donor to free-threshing wheat species, and wild barley (*Hordeum spontaneum*) are very numerous from the beginning of the occupation. While wild barley decreases in relative proportions throughout the entire sequence, goatgrass appears abruptly reduced in layers V and IV. These trends are partially reflected in the potential arable weed species. These however increase from layer III onwards together with the wheat species (*Triticum* spp.). Large-seeded pulses, such as lentil (*Lens* sp.) and vetch (*Vicia* sp.) occur in lower proportions with a peak in layer VII and then continuously decrease until layer II. Small-seeded grasses strongly increase in layers V and IV and out-number all other taxa.

**Figure 6:**
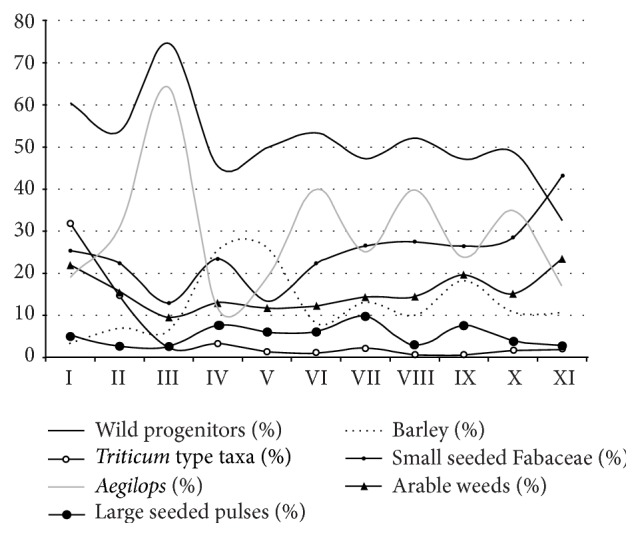
Development of the proportions of taxa groups without the small-seeded grasses.

When the dominant small-seeded grass taxa are excluded from the assemblage, the proportion patterns change (Figure 6). The most conspicuous pattern becomes visible for wild barley, which is now comparatively higher in horizons V and IV, while other large-seeded grasses, goatgrass in particular, decrease.

Presuming that the percentages of taxa groups in the different layers are representative for the subsistence development of the entire site, shifts in plant use may be evident. Notable changes are visible in layers V and IV with decreasing proportions of large-seeded grasses, mostly represented by goatgrass, and an increase in proportions of barley and small-seeded grasses.

**Figure 7:**
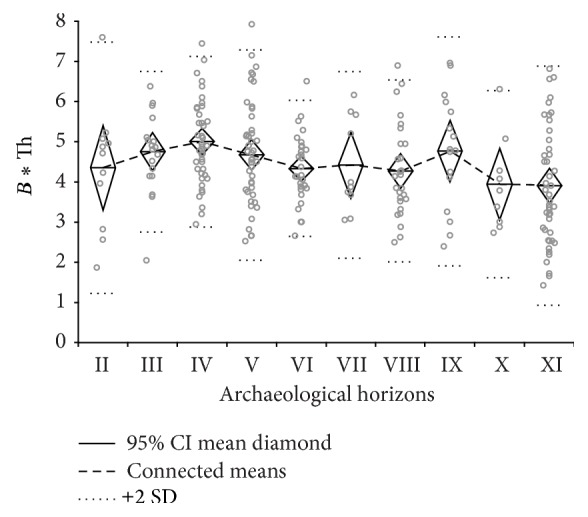
Development of barley grain sizes (*B ∗* Th: breadth multiplied by thickness) throughout the stratigraphic sequence.

Size development of barley grains has been documented throughout the archaeological horizons (Figure 7). Seed size increase in wild cereal species has been linked to increased plant management and cultivation [2]. An interesting observation is the correlation of the grain size development with the percentages of barley after exclusion of the small-seeded grasses from the assemblage. Both grain size mean values and proportion percentages of wild barley increase from layer XI to IX, then decrease and increase again in layers V and IV followed by a decrease from layer III onwards. In layer IV the first domesticated-type rachis segments of barley appear, indicating the emergence of potentially genetically changed barley cultivars by roughly 10.000 cal BP. This correlates with the increased proportions of wild barley in layers V to IV. However, the rachis internodes occur in very low proportions, consistent with the non-brittle lower rachis segments occurring in wild populations of barley as reported by Kislev [102]. Domesticated-type barley does not occur in the following layers III–I.

At the current stage of the analysis the earliest presence of unequivocally domesticated-type taxa occurs from layer II onwards with the presence of phenotypically domesticated emmer wheat chaff (*Triticum dicoccum*), corresponding with an increase of potential arable weed taxa and a decrease in goatgrass.

#### 3.1.2. The Faunal Remains

**Figure 8:**
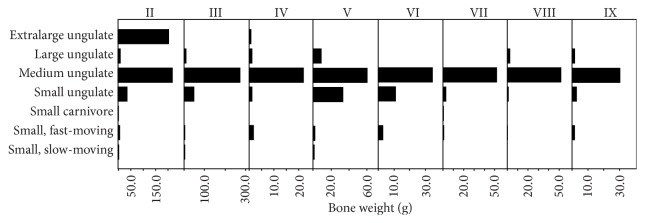
Taxa groups by bone weight for horizons with large samples (NISP > 50).

Ungulates are the most commonly identified group, followed by fish (Figure 8), which are mostly represented by small vertebrae. Among the ungulates, sheep/goat (*Ovis* or* Capra* sp.), gazelle (*Gazella gazella*), pig (*Sus scrofa*), red deer (*Cervus elaphus*), and cattle (*Bos* sp.) are most common. Other taxa include tortoise (*Testudo* sp.), hedgehog (*Erinaceus europaeus*), red fox (*Vulpes vulpes*), Eurasian lynx (*Lynx lynx*), and unidentified medium-sized birds.

Large game, in particular medium ungulates such as sheep and goat, dominate the assemblage by mass throughout the sequence. There is a spike in small ungulates (i.e., gazelles) in horizons V and IV, which correlates to the changes of the archaeobotanical assemblage. Bone weight values of extra-large ungulates (i.e., cattle) increase in horizon II, corresponding with the appearance of domesticated-type emmer chaff.

There are no temporal trends in the proportion of small to large game by mass through the sequence, nor are there changes in the small game component itself. There is, however, a statistically significant trend in the increase of cattle in later phases of the sequence (for detailed statistical results see [103]).

### 3.2. Environmental Dynamics

#### 3.2.1. Wood Charcoal

**Figure 9:**
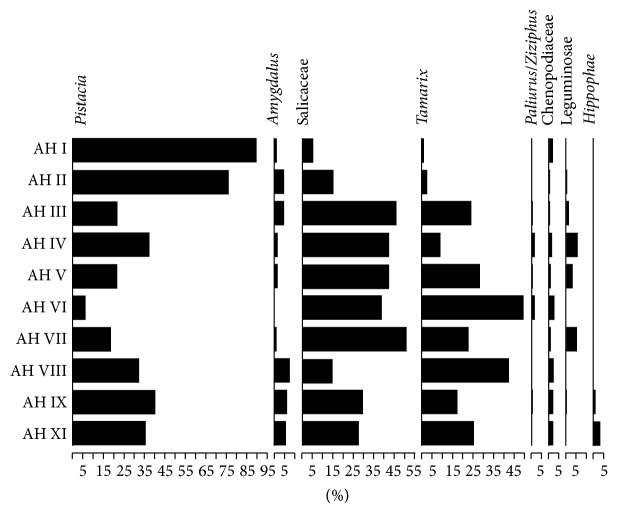
Anthracological diagram showing percentage fragment counts of the main taxa represented in the Chogha Golan deep sounding sequence (see also Table 3).

The analysis of the anthracological remains points to the existence of two major arboreal vegetation catchments in the vicinity of Chogha Golan: the semi-arid* Pistacia*-*Amygdalus* woodland and riparian vegetation habitats dominated by Salicaceae and* Tamarix* (Table 3, Figure 9). Chenopodiaceae shrubs (likely present in both zones) form a minor component of charcoal sample composition, although they are ubiquitous across the sampled sequence. Other less frequent taxa include* Paliurus/Ziziphus* and Leguminosae (subf. Papilionoideae), both of which might have been associated with riparian woodland habitats. The presence of* Hippophaë* is limited in the earliest sampled horizons (AH XI, AH IX). This taxon is considered by Zohary as tolerant of arid and cold conditions and its presence might represent a residual element from vegetation communities that were locally widespread during the Younger Dryas [66]. Other very rare taxa include* Vitex* (a riparian shrub),* Prunus* (diffuse porous; likely to represent some variety of wild cherry), Maloideae (subfamily of the Rosaceae including wild apples, pears and hawthorn),* Acer* (maple) and various shrubs (cf.* Ephedra*, cf. Labiatae). Fragments of grass stems (including charcoal particles identified as* Phragmites*) were also very occasionally present in the examined samples (not included in the wood charcoal counts). With the exception of* Vitex, Ephedra* and Lamiaceae the remaining wood charcoal taxa are likely to have derived from vegetation catchments located at some distance from the site. In any case, their rarity suggests that they were not routinely and/or intensively collected as fuel wood.

The variations observed in the ubiquity (sample presence) and frequencies (percentage fragment counts; see also Table 3, Figure 9) of individual taxa provide some useful insights in the temporal fluctuations of the intensity of use and the availability of arboreal vegetation habitats near the site. An important first observation is that* Pistacia-Amygdalus* woodland was present and routinely used as a source of wood at Chogha Golan throughout the roughly 2000 years long habitation of the site, from its earliest sampled levels dated to the last phases of the Younger Dryas to the 10th millennium cal BP.* Pistacia* and* Amygdalus* charcoals account for >35% of charcoal sample composition in AH XI, AH IX-VIII (c. 11.740–10.650 cal BP). A decrease in both taxa is observed from AH VII and is more pronounced in AH VI.* Pistacia* values pick up very quickly in AH V (rising from 6.8% in AH VI to 21.67% in AH V) and continue to increase through the remainder of the sampled sequence, reaching 90% by AH I (currently dated to c. 9.640 cal BP). Overall, the wood charcoal samples corresponding to the much shorter time period represented by AH VII–IV (10650–10040 cal BP) are dominated by riparian taxa (particularly Salicaceae; the ubiquity of Papilionoideae charcoals is also noteworthy, representing in all cases young twig fragments). The wood charcoal samples derived from the chronologically latest part of the sequence (AH III–I; c. 10040–9640 cal BP) indicate yet another shift in sample composition from AH III, still dominated by Salicaceae, to AH II-I that are overwhelmingly dominated by* Pistacia*.

#### 3.2.2. Stable Carbon Isotopes in Barley Grain

**Figure 10:**
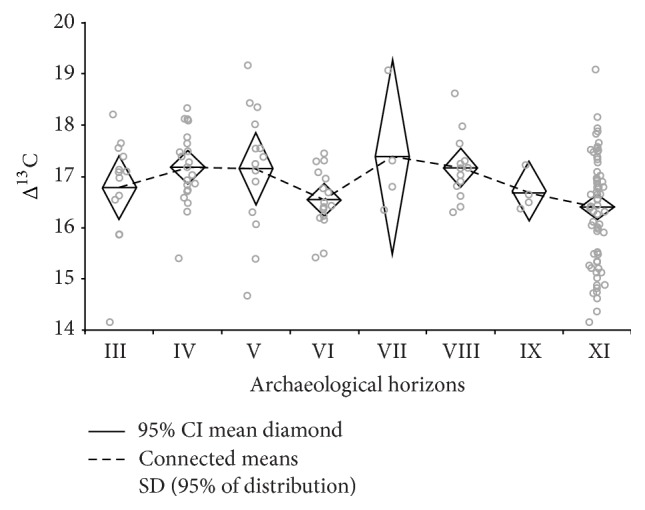
Δ^13^C measurements of wild barley grains throughout the stratigraphic sequence of Chogha Golan. Each circle represents a measurement for a single grain.

Most of the 159 measurements are available from archaeological layer XI (Figure 10). For horizons IX and VII only four measurements each could be obtained so far, while the remaining data distributes relatively equally on the other horizons. Despite the minimum target of six measurements per sample to cover the full range of variability in one sample [100], layers IX and VII show a sufficiently large range of values.

The range between 17‰ and 16‰ has elsewhere been defined to represent a transitional area between well-watered conditions for cereal growth (above 17‰) and drought stress (below 16‰) [99]. The measurements for each archaeological horizon demonstrate wide ranges, as is generally the case with increasing numbers of measurements. Most important are the mean and minimum values which represent the generalized signal (mean value) on one hand and the highest measured stress (minimum value) on the other. At Chogha Golan, there are no mean values below 16‰, indicating that drought stress was generally not a major impediment for plant growth. In some layers mean values were above 17‰, suggesting that the growing conditions for wild barley represented in layers VIII, VII, V and IV were generally under sufficient moisture availability. Archaeological layers XI, VI and III with mean values below 17‰ and minima values below 16‰ can be interpreted to show moderate drought stress signals in the plants.

Although the Δ^13^C data do not indicate extreme drought stress signals in any of the sampled horizons, they suggest fluctuations of the moisture availability with reduced moisture levels available for plant growth particularly in horizon VI, which may have resulted in the reduction of or regional shifts in some plant populations.

## 4. Discussion

### 4.1. Environmental Fluctuations and Their Possible Impact on the Living Conditions at Chogha Golan

Various reasons have been discussed as influential factors on the development of domesticated species, attributing more influence to either humans in relation to their degree of consciousness and socio-cultural needs or to fluctuating natural conditions, such as moisture and temperature increase after the Younger Dryas or increasing atmospheric CO_2_ concentration.

A major argument against multiple centers of origin of domesticated species and for the origins of domesticated plants in the Levant was formerly based on differences in vegetation development in the various geographic regions of the Fertile Crescent, in particular the later expansion of oak in the Zagros region compared to the Levant [68, 77]. Despite limited soil moisture availability in the eastern part of the Fertile Crescent, grasses expanded at the end of the Younger Dryas, suggesting that the principal resource situation as concerns the availability of grain food was relatively similar in the entire Fertile Crescent. Decreasing oxygen isotope values alongside declining Chenopodiaceae and* Artemisia* pollen as reflected in the Zeribar records that are synchronous with AH XI throughout AH IX at Chogha Golan, suggest increases in moisture. This is in agreement with the size development of barley grains and the *δ* ^13^C record from barley (Figures 7 and 10).

However, the position of Chogha Golan in a diversified region in terms of deficits or surpluses of rainfall suggests a potential for considerable environmental fluctuations in relation to inter-annual weather variability and climate change.

As C3 plants are particularly sensitive to changes in atmospheric CO_2_, the relatively low CO_2_ concentrations during the Pleistocene have been used as an explanation for why agriculture only started in the Holocene and not earlier (e.g., [23, 24, 104]). Atmospheric CO_2_ concentration during the critical sequence from large-scale gathering of wild cereals at Ohalo II around 23.000 BP increased from roughly 200 *μ*mol mol^−1^ to 270 *μ*mol mol^−1^ [105] until the end of the Younger Dryas when evidence of cultivating wild cereals started to become more frequent and remained relatively stable until approximately 1800 AD. Lower CO_2_ concentrations are associated with reduced rates of photosynthesis which often results in the decreased production of storage carbohydrates and lowered plant productivity [104]. An increase from 200 to 270 *μ*mol mol^−1^ stimulates photosynthesis and biomass productivity of C3 plants by 25–50% which served as the main argument to explain the worldwide beginnings of agriculture only in the Holocene [23]. If we assume that this model is possible, we may further ask whether fluctuations in atmospheric CO_2_ in the early Holocene may have resulted in extended phases of lower biomass productivity thus protracting the evolution of domesticated species.

The atmospheric concentration of CO_2_ effects the water balance of C3 plants, because increased CO_2_ reduces the stomatal aperture, thus lowering transpiration. This means that increasing CO_2_ towards the end of the Pleistocene could have led to reductions in water consumption, reducing the intensity of drought stress experienced by the plants [23]. This has a theoretical impact on our interpretation of the Δ^13^C values in barley from Chogha Golan, as fluctuations toward decreased CO_2_ could produce relative stress signals in the Δ^13^C record that are indicative of the short-term lowering of CO_2_ concentration rather than for a reduction in water availability, as has been interpreted for the Δ^13^C values in barley from AH VI. However, this assumption is hypothetical as research on low CO_2_ concentrations and vegetation responses are still very limited.

The Chogha Golan sequence falls into the early Holocene with CO_2_ concentrations around 270 *μ*mol mol^−1^ and fluctuations in CO_2_—for example, indicated in reconstructions of carbon-cycle dynamics based on trapped CO_2_ at Taylor Dome [106]—may have influenced the productivity of the vegetation on a local and regional scale. In particular the data from the Byrd Station ice core indicate relatively low CO_2_ concentrations around 10.000 BP which may be related to the low Δ^13^C values in AH VI. However, using raw data available from the World Data Center for Paleoclimatology, Boulder [107], lower CO_2_ values occur towards the end of the occupation period at Chogha Golan, but are not chronologically related to AH VI. Given the relatively stable development of CO_2_ concentrations between 11.800 and 10.000 BP, the lowered Δ^13^C values from Chogha Golan in horizon VI seem to support a moderate drought stress signal.

A peak in salt-tolerant* Tamarix* charcoal and the pronounced decrease of* Pistacia* and* Amygdalus* that may have formed open woodland communities as observed in AH VI are consistent with the interpretation of slight drought stress, but could alternatively indicate a reduction in biomass productivity in relation to lower CO_2_ concentrations.

Integrating the Lake Zeribar palaeoenvironmental record into our data sets we may delineate the following environment-subsistence-coupled developments.

Before the earliest occupation of the site, temperatures increased with the end of the Younger Dryas. High evaporation and low precipitation led to low lake levels, water salinization and scattered occurrence of a very thin arboreal cover, dominated by Chenopodiaceae. During the time of the first settlement horizon (AH XI), conditions were still relatively cold and dry, as indicated by the occurrence of* Hippophaë* charcoal, a moderate drought stress for wild barley at Chogha Golan, and high Chenopodiaceae and* Artemisia* pollen values at Lake Zeribar. At the same time* Pistacia* charcoal as well as grains of barley and goatgrass are already present in relatively high proportions and grain size mean values of barley increase, indicating intensive plant management. Environmental conditions changed until AH VIII when decreased Chenopodiaceae and* Artemisia*, and increased Poaceae pollen are indicated at Lake Zeribar which have been interpreted as reduced summer drought. Changes in seasonality resulted in the dominance of Poaceae pollen and an initial increase in arboreal pollen, represented by a* Pistacia*-deciduous oak semi-arid grassland. These shifts are reflected in abundant* Pistacia* charcoal at Chogha Golan and sufficient moisture availability for wild barley. However, grain size mean values and proportional percentages of wild barley decrease in AH VIII, suggesting some limitations in the use of barley which may have been compensated by goatgrass. In AH VII a few dung fragments and coprophilous fungi have been observed, while for the following sequence (AH VI) vegetation disturbance by hunters and/or herders has been suggested for the Lake Zeribar region. For this horizon the lowest* Pistacia* charcoal proportions have been documented, while Salicaceae and* Tamarix* percentages are increased. Drought and/or lowered biomass productivity may have limited the efficiency of plant management, which might also have resulted in a temporary change in animal prey availability. The latter becomes apparent in AH V with increased bone weight for gazelle. This is accompanied by large numbers of small-seeded grasses, a strong reduction in goatgrass and a relative peak in wild barley that may have been intensively cultivated under sufficient moisture availability in relation to the previous shortcomings in AH VI. Evidence for the intensified cultivation of wild barley is supported by increased grain size mean values. The conditions are unchanged in AH IV when first domesticated-type rachis segments of barley appear alongside a possible clay animal head.

In AH III the first evidence for the use of dung fuel is represented in the remains of a floor and suggests the intentional collection of dung for burning or construction, or increased access to dung from keeping certain species at the site. In this layer, grain size mean values and proportional percentages of wild barley decrease, whereas in the following layer AH II an increase of arable weed taxa and wheat species is apparent.* Pistacia* pollen increases in a relative sense and oak pollen is also present at Lake Zeribar. This correlates with wood charcoal finds from Chogha Golan which show a steep increase in* Pistacia* percentages. In this horizon the first domesticated-type emmer chaff and an increase in cattle bone is documented. The first animal figurines representing cattle also occur in this horizon, as well as obsidian that comprises 0.5% of the lithic material. In AH I* Pistacia* charcoal increases further, and mortars, pestles and grinding slabs are frequent. Overall, the environment-subsistence-coupled developments suggest that despite the moderate fluctuations in biomass productivity for various reasons, the inhabitants of Chogha Golan created resilient living conditions through an increase in the use of large-bodied ungulates and cereal cultivation.

### 4.2. Changes in Resource Availability or Limitations to the Recognition of Spatial Patterns?

Although the main seed and faunal taxa groups from Chogha Golan are relatively stable in composition throughout the archaeological layers, changing proportions of taxa in the middle (AH V and IV) and the late (AH II) parts of the sequence are considerable. While most of the archaeological horizons are dominated by large-seeded grasses, that is, goatgrass and barley, layers V and IV contain large amounts of small-seeded grasses and show signs of intensification of barley cultivation (Figure 6). The first domesticated-type rachis segments of barley and a relative seed size increase appear in layer IV. Resource stress, following on somewhat drier conditions for plant growth at the time of the formation of horizon VI, might have been present, which is corroborated by an increase in gazelle bones. The general lack of diachronic changes in the ratio of proportions of easy to procure small, slow-moving animals and more difficult to catch small, fast-moving game might be interpreted as an absence of resource stress [91, 95, 108, 109] (see [103] for an in-depth discussion of small game at Chogha Golan), though this might also reflect the small sample size of the current assemblage. There is also no significant change in the charcoal assemblages that are dominated by Salicaceae in AH V and IV. In general, AH VII–III are dominated by riparian taxa, while* Pistacia* (and to a lesser degree* Amygdalus*) register high values (percentage frequencies and ubiquity) in AH I-II.* Pistacia* and* Amygdalus* also have high values in the earlier part of the sampled sequence (AH XI–VIII). It is therefore possible that a different spatial pattern of fuel use and deposition of fuel debris is reflected in charcoal sample composition from AH VII–III (e.g., opportunistic clearance and use of riparian vegetation by mobile herders/hunters).

Preliminary micromorphological analyses have indicated that middens, as revealed throughout AH VII–III were likely accumulated inside abandoned structures [110]. Although there is no evidence to suggest that any of the sampled levels in the deep sounding could have been used as an animal penning area, very few dung fragments and coprophilous fungi were observed in AH VII [110, p. 129]. Small-seeded grasses and pulses are often interpreted as potential forage for herbivore species grazing in late summer. While the small-seeded grasses have very high frequencies in AH V and IV, small-seeded pulses are decreased by comparison in AH V, but increase in AH IV. It is possible that these levels represent a spatial pattern in the use of plant-derived resources compared to areas that are more likely to contain the debris of domestic wood fuel. Although dung spherulites were occasionally observed throughout the studied sequence, the first definitive evidence for the use of dung fuel derives from the remains of a floor preserved in AH III [110]. It is therefore possible that dung was used, alongside wood, as a complementary source of fuel or for the production of construction materials. However, the available evidence (including anthracological, seed botanical, micromorphological and stratigraphic data) does not support increasing use of dung fuel through time as a substitute for depleted woodland sources, but rather points to the resilient nature of the local landscape and its resources.

A major concern in interpreting our data is the question of the representativeness of a 1 m² excavation area, and whether the fluctuations described above, particularly in AH V and IV, indeed indicate a shift in resource use or subsistence, or if the patterns only reflect the limitations of the excavation. Synchronous shifts in the proportions of some important plant and animal taxa, the correlation of barley proportions with grain size development, as well as the appearance of domesticated-type barley rachis internodes in AH IV and domesticated-type emmer chaff in AH II support the patterns as representative. The wood charcoal data, however, do not correlate with the changes in the seed and faunal assemblages. Instead they are in relatively good agreement with the Δ^13^C values in barley.

**Figure 11:**
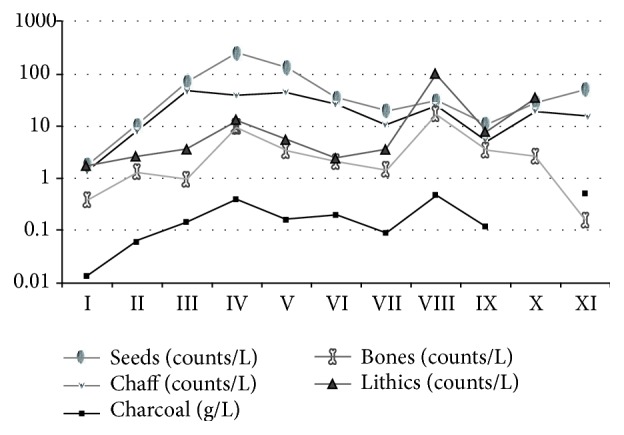
Find densities of the different find groups over the stratigraphic sequence.

Find densities of the different find groups may partially clarify the complex relationship of taphonomy and contextual differences in the different materials recovered from the deep sounding (Figure 11).

The general trend of the find densities is very similar in all of the groups and may indicate underlying contextual aspects of the deposits. Except for animal bones, find densities of the other categories are high in AH XI and decrease until AH IX, though densities of faunal remains must be examined with caution due to the differential wet screening methods mentioned above. Charcoal, animal bones and lithic artefacts show their peak in find density in AH VIII. The densities of all find categories decrease after AH VIII, stagnate or slightly increase in AH VI and V, and again peak in finds in AH IV. After AH IV there is a continuous decrease in find densities in all categories, except for chaff remains, indicating their intense production in the settlement. In AH I the find densities are particularly low as a result of taphonomic processes in close proximity to the soil surface.

As concerns the specific question of AH V and IV reflecting some kind of bottleneck in subsistence, the high find densities in all categories suggest that a bias through selective preservation of the more fragile organic remains can be excluded (i.e. the reduced presence of large-seeded grasses and increased weights of gazelle bones very likely reflect the contextual characteristics of these layers).

## 5. Conclusions

The long-term subsistence reliance on cultivated wild plant species and wild game, with the first perpetual domesticated-type emmer appearing after almost 2.000 years of occupation suggests, despite a short-term depression in horizons V and IV, a generally proliferous environment, rich in resources at Chogha Golan.

The development of barley through time supports the model of protracted domestication. Both proportions and grain sizes of wild barley increase from AH XI to IX and correlate with Δ^13^C signals of increasing moisture availability. The slight drought stress signal in AH VI, which can be easily understood as a plausible effect of inter-annual variation of precipitation, is followed by changes in the plant and faunal assemblages in layers V and IV. These layers show increased proportions of small-seeded grasses and gazelle bone, and a relative increase of wild barley proportions and its grain size. In AH IV, roughly around 10.200 cal BP barley rachis internodes also show domesticated-type features that disappear again in the following horizons. The short-term appearance of domesticated-type characteristics in barley suggest that the inhabitants at Chogha Golan were about to domesticate barley around 10.200 cal BP, but continued with the earlier practice of plant use (i.e. probably introducing again more wild types). Grain size and Δ^13^C signals decrease again in AH III. With the transition to AH II further changes become apparent that include an increase in* Triticum* species, including phenotypically domesticated emmer wheat chaff (*Triticum dicoccum*), increasing percentages of potential arable weeds and increasing numbers of cattle bones.

In our previous work about Chogha Golan we argued that domestication of emmer may have evolved on-site [4], supported by the long tradition in cultivating a broad range of wild progenitor species of modern crops at the site, including the occurrence of domesticated-type barley rachis internodes non-recurring in layer IV, and biomolecular evidence of a separate genepool in this area [111, 112]. There are, however, models suggesting the dispersal of wild and/or domesticated emmer from elsewhere [113] that are included in earlier archaeological hypotheses on transport of cereal grain into regions outside their original area of cultivation [114] and supported by obsidian trade from southern Anatolia into our study region [60].

Hypotheses concerning the transport of grain from elsewhere, such as the western Fertile Crescent, can indeed be used to explain a protracted development of domesticated species. However, this argument is less convincing in a proliferous environment offering sufficient resources, and thus enabling a resilient way of living. Given the fact that there is evidence for the extensive use of plants, we may question whether the development of agriculture really required significant changes in human subsistence behavior or whether these changes were gradual and appear to be substantial only from our modern perspective.

Overall, the entire stratigraphic sequence at Chogha Golan can be seen as a continuously increasing intensification in the use of grasses, even in layers V and IV. However, a bottleneck in the availability of large-seeded grasses might have occurred during the formation of these two strata, which was attenuated by the increased gathering of small-seeded grasses, shift to small-bodied ungulates and intensified cultivation of barley, followed by emmer cultivation in the subsequent phases.

## Figures and Tables

**Table 1:** Radiocarbon AMS data from Chogha Golan. Dating has been conducted at the AMS laboratory of the Universities of Erlangen, Uppsala, and Kiel. Calibration BP with *calPal-online.de*, BC 2*σ* after Reimer et al. [64].

Species/archaeological horizon	Lab code	BP	Cal BP	Cal BC (2*σ*)
*Hordeum spontaneum*, geological horizon	KIA45647	9330 ± 35 (base residue) 10125 ± 45 BP (humic acid)	10549 ± 37 (b.r.) 11740 ± 187 (h.a.)	8658–8529 (b.r.) 10042–9651 (h.a.)

*Hordeum spontaneum*, AH XI	KIA44943	9790 + 120/−110	11162 ± 220	9556–8812

*Hordeum spontaneum*, AH XIa	KIA45648	9320 ± 120 BP (base residue) 10230 ± 45 BP (humic acid)	10527 ± 166 (b.r.) 11952 ± 137 (h.a.)	8847–8283 (b.r.) 10155–9817 (h.a.)

*Hordeum spontaneum*, AH XI	KIA44944	9690 ± 45	11054 ± 129	9274–9119

*Hordeum spontaneum*, AH XI	KIA44942	9385 ± 37 (base residue) 9590 ± 40 (humic acid)	10622 ± 45 (b.r.) 10945 ± 123 (h.a.)	8754–8565 (b.r.) 9118–8872 (h.a.)

*Hordeum spontaneum*, AH VIII	KIA43836	9425 ± 45	10656 ± 53	8814–8602

*Hordeum spontaneum*, AH VI	Ua-44324	8812 ± 53	9910 ± 157	8117–7803

*Aegilops* sp., AH V	Ua-44323	8845 ± 54	9954 ± 148	8152–7856

*Hordeum spontaneum*, AH IV	Erl-14839	8887 ± 37	10037 ± 94	8234–7938

Poaceae, AH III	Erl-14840	8805 ± 38	9839 ± 81	8181–7731

*Hordeum spontaneum*, AH III	Erl-14838	8770 ± 40	9788 ± 84	7967–7648

*Aegilops* glume bases, AH II	KIA45649	8965 ± 35	10091 ± 107	8286–8181

Cerealia, AH II	KIA44941	9518 ± 44	10887 ± 147	8934–8713

*Hordeum spontaneum*, AH II	Beta336511	9110 ± 40	10319 ± 59	8460–8280

*Triticum* type species, AH II	Beta336510	8800 ± 40	9831 ± 82	8020–7750

Charcoal, AH I	Beta336509	8900 ± 40	10045 ± 95	8240–7950

Charcoal, AH I	Beta336508	8690 ± 40	9637 ± 54	7790–7600

**Table 2:** Archaeobotanical taxa (seeds or fruits unless otherwise stated) and their overall find number, proportions, and frequencies in the deep sounding; ^·^ <0,01%, ^•^ <0,1%, and ^#^ <1%.

Family	Taxa	Σ	Proportion in %	Frequency in %
Amaranthaceae	*Amaranthus* sp.	2	•	18
Amaranthaceae	*Atriplex* cf. *prostrata* (fruit and perianth)	23	#	55
Amaranthaceae	*Suaeda* sp.	61	#	45
Anacardiaceae	*Pistacia* sp.	518	2	100
Anacardiaceae	*Pistacia terebinthus *	2	•	9
Apiaceae	Apiaceae indet.	1	·	9
Asteraceae	*Anthemis cotula *	1	·	9
Asteraceae	Asteraceae indet.	1	·	9
Asteraceae	*Centaurea* sp.	79	#	91
Asteraceae	cf. *Carthamus* sp.	1	·	9
Boraginaceae	*Arnebia* sp.	6	•	27
Boraginaceae	Boraginaceae indet.	4	•	27
Boraginaceae	*Heliotropium* sp.	94	#	100
Boraginaceae	*Lithospermum arvense*, uncarbonized	16	#	73
Boraginaceae	*Lithospermum tenuiflorum*, uncarbonized	2	•	9
Brassicaceae	*Alyssum* sp.	2	•	18
Brassicaceae	Brassicaceae indet.	275	1	100
Brassicaceae	*Lepidium/Sisymbrium *	2	•	18
Capparidaceae	*Capparis* sp.	1	·	9
Caryophyllaceae	*Arenaria* sp.	1	·	9
Caryophyllaceae	Caryophyllaceae indet.	114	#	91
Caryophyllaceae	*Gypsophila elegans* type	1	·	9
Caryophyllaceae	*Gypsophila* sp.	39	#	55
Caryophyllaceae	*Silene* sp.	118	#	91
Caryophyllaceae	*Silene/Arenaria *	3	•	9
Chenopodiaceae	*Beta vulgaris *	1	·	9
Chenopodiaceae	Chenopodiaceae/Amaranthaceae	52	#	36
Chenopodiaceae	*Chenopodium* sp.	18	#	64
Chenopodiaceae	*Salsola kali *	28	#	73
Convolvulaceae	*Convolvulus* type	2	•	18
Cyperaceae	*Carex* sp.	1	·	9
Cyperaceae	Cyperaceae indet.	5	•	9
Cyperaceae	*Scirpus* cf. *maritimus *	77	#	91
Cyperaceae	*Scirpus* sp.	74	#	91
Fabaceae	*Astragalus* sp.	1764	6	100
Fabaceae	cf. *Lathyrus* sp. (cylindric)	12	•	18
Fabaceae	cf. *Trigonella* sp.	17	#	9
Fabaceae	*Coronilla* sp.	50	#	55
Fabaceae	*Coronilla/Trigonella *	13	•	9
Fabaceae	Fabaceae indet., large	1	·	9
Fabaceae	Fabaceae indet., medium	55	#	64
Fabaceae	Fabaceae indet., small	1396	4	100
Fabaceae	*Galega/Ornithopus* type	4	•	9
Fabaceae	*Lathyrus/Pisum/Vicia *	317	1	100
Fabaceae	*Lathyrus/Vicia *	124	#	55
Fabaceae	*Lens* sp.	455	1	100
Fabaceae	*Medicago radiata *	103	#	91
Fabaceae	*Medicago* sp.	72	#	27
Fabaceae	*Pisum* sp.	2	•	9
Fabaceae	*Trifolium* type	1	·	9
Fabaceae	*Trigonella* sp.	2222	7	100
Fabaceae	*Trigonella/Astragalus *	37	#	9
Fabaceae	*Vicia* type	3	•	18
Geraniaceae	*Erodium *sp.	19	#	9
Lamiaceae	Lamiaceae indet.	1	·	9
Lamiaceae	*Ocimum/Salvia *	1	·	9
Liliaceae	*Ornithogalum/Muscari *	100	#	91
Malvaceae	cf. *Malva* sp.	258	1	100
Papaveraceae	*Fumaria* sp.	3	•	9
Papaveraceae	*Papaver* sp.	28	#	9
Plantaginaceae	*Veronica opaca* type	1	·	9
Poaceae	*Aegilops* sp.	139	#	91
Poaceae	*Aegilops* sp., glume base	4698	15	100
Poaceae	*Aegilops/Hordeum *	19	#	55
Poaceae	*Aegilops/Triticum*, glume base	18	#	18
Poaceae	*Agrostis* sp.	4	•	9
Poaceae	*Avena fatua *type	56	#	18
Poaceae	*Bromus *type	87	#	82
Poaceae	*Bromus/Brachypodium *	46	#	36
Poaceae	Cerealia	3	•	9
Poaceae	*Dasypyrum*-type	18	#	9
Poaceae	*Echinaria capitata *	1	·	9
Poaceae	*Eragrostis* sp.	201	1	55
Poaceae	*Eremopyrum* sp.	1	·	9
Poaceae	*Hordeum* cf.* Spontaneum *	444	1	91
Poaceae	*Hordeum distichum*, rachis	25	#	45
Poaceae	*Hordeum distichum* type	17	#	64
Poaceae	*Hordeum* sp.	227	1	100
Poaceae	*Hordeum* sp., rachis	479	2	100
Poaceae	*Hordeum spontaneum*, rachis	1698	5	91
Poaceae	*Hordeum/Taeniatherum *	34	#	18
Poaceae	*Phalaris* sp.	1278	4	100
Poaceae	*Phleum *type	51	#	36
Poaceae	Poaceae indet., awn fragments	47	#	9
Poaceae	Poaceae indet., large	606	2	100
Poaceae	Poaceae indet., large, glume base fragments	997	3	100
Poaceae	Poaceae indet., medium	670	2	73
Poaceae	Poaceae indet., small	10372	33	100
Poaceae	*Secale* type	13	•	9
Poaceae	*Stipa* type	45	#	18
Poaceae	*Taeniatherum caput-medusae/crinitum *	67	#	73
Poaceae	*Taeniatherum caput-medusae/crinitum*, rachis	110	#	64
Poaceae	*Triticum boeoticum/dicoccoides*, glume base	28	#	64
Poaceae	*Triticum boeoticum/dicoccoides *	49	#	73
Poaceae	*Triticum* cf. *boeoticum *	27	#	9
Poaceae	*Triticum* cf. *boeoticum* (*thaoudar*, 2-grained)	10	•	9
Poaceae	*Triticum* cf. *dicoccum*, glume base	25	#	18
Poaceae	*Triticum* cf. *monococcum*, 1-grained	26	#	9
Poaceae	*Triticum* sp. glume base	205	1	82
Poaceae	*Triticum* sp.	10	•	36
Poaceae	*Triticum*, free-threshing type rachis	3	•	18
Poaceae	Triticoid type (acc. Van Zeist)	169	1	91
Polygonaceae	*Rumex/Polygonum *	4	•	9
Ranunculaceae	*Adonis* sp.	11	•	45
Resedaceae	*Reseda luteola *	8	•	9
Rosaceae	*Prunus* sp., fragment	1	·	9
Rubiaceae	*Galium* sp.	36	#	73
Solanaceae	*Solanum* sp.	1	·	9
Vitaceae	cf. *Vitis* sp.	1	·	9
	Indet.	53	#	45
	Ostracoda cf. Candona sp.	14	•	45

**Table 3:** Quantified anthracological data from Chogha Golan (deep sounding; horizons AH XI, IX–IV, and III–I) tabulated by absolute fragment counts (*C*), percentage fragment counts (%), and sample presence (*U*).

	XI			IX			VIII			VII			VI			V			IV			III			II			I		
	*C*	%	*U*	*C*	%	*U*	*C*	%	*U*	*C*	%	*U*	*C*	%	*U*	*C*	%	*U*	*C*	%	*U*	*C*	%	*U*	*C*	%	*U*	*C*	%	*U*
*Pistacia *	32	35.56	4	72	40.45	4	13	32.50	2	17	18.48	4	9	6.38	4	39	21.67	4	45	37.50	2	28	21.88	4	114	76.00	7	81	90.00	8
*Amygdalus *	5	5.56	2	11	6.18	3	3	7.50	2	1	1.09	1				3	1.67	2	2	1.67	1	6	4.69	3	7	4.67	4	1	1.11	1
*Prunus *													1	0.71	1															
cf. Maloideae																1	0.56	1												
*Acer *				1	0.56	1																								
Salicaceae	25	27.78	4	53	29.78	4	6	15.00	2	47	51.09	4	55	39.01	4	76	42.22	4	51	42.50	2	59	46.09	4	23	15.33	4	5	5.56	3
*Tamarix *	23	25.56	4	31	17.42	4	17	42.50	2	21	22.83	4	70	49.65	4	51	28.33	4	11	9.17	2	31	24.22	4	4	2.67	4	1	1.11	1
*Hippophae *	3	3.33	2	2	1.12	2	0																							
Chenopodiaceae	2	2.22	2	4	2.25	1	1	2.50	1	1	1.09	1	4	2.84	3	2	1.11	2	2	1.67	1	1	0.78		1	0.67	1	2	2.22	1
*Paliurus/Ziziphus *				1	0.56	1							2	1.42	2	1	0.56	1	2	1.67	2	1	0.78	1						
*Vitex *				1	0.56	1																								
Leguminosae				1	0.56	1				5	5.43	2				6	3.33	3	7	5.83	2	2	1.56	2	1	0.67	1			
cf. *Ephedra *																1	0.56	1												
cf. Labiatae				1	0.56	1																								

Total	90	100	(*n* = 4)	178	100	(*n* = 4)	40	100	(*n* = 2)	92	100	(*n* = 4)	141	100	(*n* = 4)	180	100	(*n* = 4)	120	100	(*n* = 2)	128	100	(*n* = 4)	150	100	(*n* = 7)	90	100	(*n* = 8)

**Table 4:** Taxa categories based on body size and predator evasion tactics. Weight ranges from Nowack [96] and Silva and Downing [97].

	Weight range (kg)
Very large ungulate	
Aurochs (*Bos primigenius*)	500–1000
Large ungulate	
Red deer (*Cervus elaphus*)	75–340
Wild pig (*Sus scrofa*)	50–350
Medium ungulate	
Persian fallow deer (*Dama mesopotamica*)	50–120
Wild goat (*Capra aegagrus*)	40–90
Wild sheep (*Ovis* sp.)	40–90
Small ungulate	
Gazelle (*Gazella gazella*)	17–23
Small carnivore	
Eurasian lynx (*Lynx lynx*)	8–38
Red fox (*Vulpes vulpes*)	8–10
Wild cat (*Felis silvestris*)	3–8
Small fast-moving	
Cape hare (*Lepus capensis*)	1.3–7.0
Rock partridge (*Alectoris graeca*)	0.51–0.68
Fish (*Pices*)	
Small slow-moving	
Tortoise (*Testudo* sp.)	1-2+
